# Association between symptoms of depression and inflammatory parameters in people aged over 90 years

**DOI:** 10.1186/s12877-024-04895-5

**Published:** 2024-04-04

**Authors:** Paulina Zabielska, Małgorzata Szkup, Artur Kotwas, Karolina Skonieczna-Żydecka, Beata Karakiewicz

**Affiliations:** 1 Subdepartment of Social Medicine and Public Health, Department of Social Medicine, Pomeranian Medical University in Szczecin, Żołnierska 48, 71-210 Szczecin, Poland; 2 Department of Social Nursing, Pomeranian Medical University in Szczecin, Żołnierska 48, 71-210 Szczecin, Poland; 3 Independent Research and Biostatistics Laboratory, Department of Social Medicine, Pomeranian Medical University in Szczecin, Żołnierska 48, 71-210 Szczecin, Poland; 4 Department of Biochemical Research, Pomeranian Medical University in Szczecin, Broniewskiego 24, 71-460 Szczecin, Poland

**Keywords:** Inflammation, Depression, Anti-inflammatory, Longevity, GDS

## Abstract

**Background:**

Old age is a period of life that presents many health and social challenges, resulting in a greater vulnerability to the development of mental disorders, including depression. There has been a growing interest in the relationship between depression and inflammatory factors, because of its potential clinical and therapeutic implications. Inflammatory processes, which were originally understood as a response to infection and trauma, appear to play an important role in the pathogenesis of depression in the elderly. The aim of the study was to analyse the relationship between the severity of depressive disorders and inflammatory parameters in people over 90 years of age.

**Methods:**

The study was conducted in Poland in 2017. The study population consisted of long-lived individuals, both living at home and staying in residential care homes. The participants were 90 people of both sexes (69 women and 21 men), aged between 90 and 103 years (mean = 92.36; SD = 2.98). The study was conducted using the diagnostic survey method with a questionnaire technique. The Geriatric Depression Scale (GDS) was used to carry out the analysis. The levels of selected inflammatory factors were determined using relevant laboratory tests.

**Results:**

In the study group, the highest percentage of people had symptoms of moderate depression (*n* = 36;40%), followed by those without depression (*n* = 35;38.9%). There was a weak negative correlation between GDS scores and fibrinogen levels (*p* ≤ 0.05). The logistic regression model showed no significant relationship between inflammatory parameters and the development of depressive disorders.

**Conclusions:**

Inflammatory parameters do not appear to predict the development of depressive disorders in people over 90 years of age.

## Background

Old age is a period of life that presents many health and social challenges, resulting in a greater vulnerability to the development of mental disorders, including depression. Depression is one of the most common psychiatric disorders affecting people over the age of 60. The health burden of depression is expected to continue to increase in the future, with the number of people aged 60 and older projected to rise from 901 million in 2015 to 1.4 billion in 2030 [1, 2]. In recent years, depression has accounted for 5.7% of Years Lived with Disability (YLDs) in people over 60 years of age [3]. Depression in old age is a major health problem because of its negative impact on quality of life, the progression of other diseases and rising healthcare costs. Older people with depression have been found to be at higher risk of suicide [4], pneumonia [5], stroke [6] and chronic obstructive pulmonary disease [7].

There has been a growing interest in the relationship between depression and inflammatory factors, because of its potential clinical and therapeutic implications. Inflammatory processes, which were originally understood as a response to infection and trauma, appear to play an important role in the pathogenesis of depression in the elderly. The immune system is thought to play an important role in the pathophysiology of psychiatric disorders in adults [8]. In patients with depression, the inflammatory response system activates the hypothalamic-pituitary-adrenal axis, leading to the production of corticotropin-releasing hormone and adrenocorticotropic hormone, as well as an increase in the turnover of serotonin and catecholamines [9]. The inflammatory response system is stimulated by pro-inflammatory cytokines secreted by macrophages, T cells and Natural killer cells (NK cells) in response to the activation of the immune system [10]. The psycho-neuro-inflammatory theory is supported by past research, with studies showing that stimulation of the hypothalamic-pituitary-adrenal axis leads to the release of corticotropin by pro-inflammatory cytokines such as interleukin (IL)-1, IL-6 and tumour necrosis factor (TNF-α) [11]. Since then, many studies have demonstrated increased levels of acute-phase proteins and pro-inflammatory cytokines such as C-reactive protein (CRP), IL-1, IL-1β, IL-6 and TNF-α in depression [12–15]. Elevated levels of peripheral IL-1β, IL-6 and TNF-α may be potential biomarkers of vulnerability to psychiatric disorders in adults, but their role in older people remains incompletely understood. In addition, reduced levels of anti-inflammatory cytokines, such as IL-10, and Transforming growth factor beta 1 (TGF-β1), have been observed in the plasma of depressed patients [16–19]. It has been also observed that the concentration of fibrinogen exhibits a statistically significant positive correlation with depressive symptoms [20].

Ageing of the human body is a process that naturally promotes the pro-inflammatory state by disrupting the peripheral immune system, leading to an overactive innate immune system with the release of pro-inflammatory cytokines and reduced levels of anti-inflammatory molecules. As the elderly population grows, understanding the mechanisms by which inflammatory factors affect the psyche and functioning of older people is becoming increasingly important.

The aim of the study was to analyse the relationship between the severity of depressive disorders and inflammatory parameters in people over 90 years of age.

## Materials and methods

### The study group

The study was conducted in Poland in 2017, in two voivodeships– West Pomerania and Masovia. The population of Masovia is largely indigenous to Poland, whereas West Pomerania is characterised by a population with a migrant background, due to the resettlements after 1945. The study population consisted of long-lived individuals, both living at home and staying in residential care homes. The study included 90 people of both sexes (69 women and 21 men) aged between 90 and 103 years (mean = 92.36; SD = 2.98). Women accounted for 76.7% of the studied population (mean age = 92.59; SD = 3.15), and men 23.3% (mean age = 92.59; SD = 2.20).

**Fig. 1:**
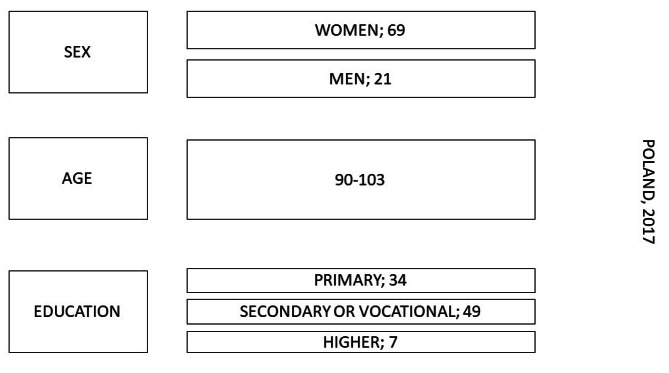
Sociodemographic data

The majority of respondents had secondary or vocational education (49), 34 respondents had primary education, and 7 respondents had higher education (Fig. 1).

### Measures

The study was based on a diagnostic survey, delivered using a face-to-face survey technique. The prevalence of depression in the study group was assessed using a screening test, the 15-item Geriatric Depression Scale (GDS). Those who scored 0–5 points were considered to be free of depression, 6–10 points indicated moderate depression and 11–15 points indicated major depression [20].

Venous blood was collected from each of the participants, after overnight fasting, between 7.00 a.m. and 9.30 a.m. in the morning, after a 10-min rest in a sitting position, from the antecubital vein using Vacutainer tubes (Sarstedt, Germany), separately into two tubes: one with 1 g/L K2 EDTA and the other for biochemical analysis of serum (7.5 mL).

The blood was collected in accordance with the relevant regulations and procedures for the collection, storage and transport of biological material. Plasma levels of proinflammatory IL-1α, IL-6, TNF-α, fibrinogen, CRP, and anti-inflammatory IL-10, TGF-β1 were measured by immune-enzymatic assays using commercially available enzyme-linked immunosorbent assay (ELISA) kits, according to the manufacturer’s protocol: DRG, Germany, for IL-1α,IL-1ß,IL-6, TNF-α, CRP, IL-10, TGF-β1 and Assaypro, USA, for fibrinogen.

All patients were thoroughly informed about the scope and objectives of the study and gave their written consent to participate. The study was approved by the Bioethics Committee of the Pomeranian Medical University (no KB-0012/47/16).

### Statistical analysis

Statistical analysis was performed using the MedCalc statistical software version 19.1.5. (Ostend, Belgium). First, the Shapiro-Wilk test for normality was applied and, based on its results, non-parametric tests were then used. Pearson’s chi-squared test was used to compare groups in terms of the distribution of nominal variables. Mann-Whitney or Kruskall-Wallis tests were used to identify associations between selected quantitative variables and nominal parameters, but only when the number of observations exceeded 5. Correlation analyses were performed using Spearman’s rank correlation method. The cut-off value for statistical significance was set at *p* < 0.05, and *p* < 0.1 was considered to indicate a statistical trend. To control type I errors, the false discovery rate (FDR) approach was used. The tables were made in APA format.

## Results

Basic descriptive characteristics are presented in Table 1.

**Table 1:** Elements of statistical description of analysed variables

Inflammatory factors	N	min-max	Mean ± SD	Median	Q1– Q3
TNF_α (pg/mL)	90	1.23–69.06	10.92 ± 10.31	8.48	6.34–12.08
TGF_β1 (pg/mL)	90	8565.00–126180.00	39293.63 ± 17163.26	37200.00	29328.00–46650.00
CRP (mg/L)	90	0.74–46.29	10.38 ± 10.16	7.31	2.96–12.88
Fibrinogen (µg/mL)	90	4.19–41.22	39.71 ± 6.47	40.89	40.81–41.05
IL_10 (pg/mL)	90	1.60–631.70	21.94 ± 68.91	2.79	1.60–19.77
IL_1α (pg/mL)	90	1.10–23.26	1.58 ± 2.52	1.10	1.10–1.10
IL_1β (pg/mL)	90	0.35–1355.00	43.15 ± 185.61	0.35	0.35–0.35
IL_6 (pg/mL)	90	2.00–668.80	85.23 ± 149.87	22.36	11.78–54.24

Q1– First Quartile; Q3– Third Quartile; N- number of population; min-minimum; max-maximum; SD-standard deviation

**Fig. 2:**
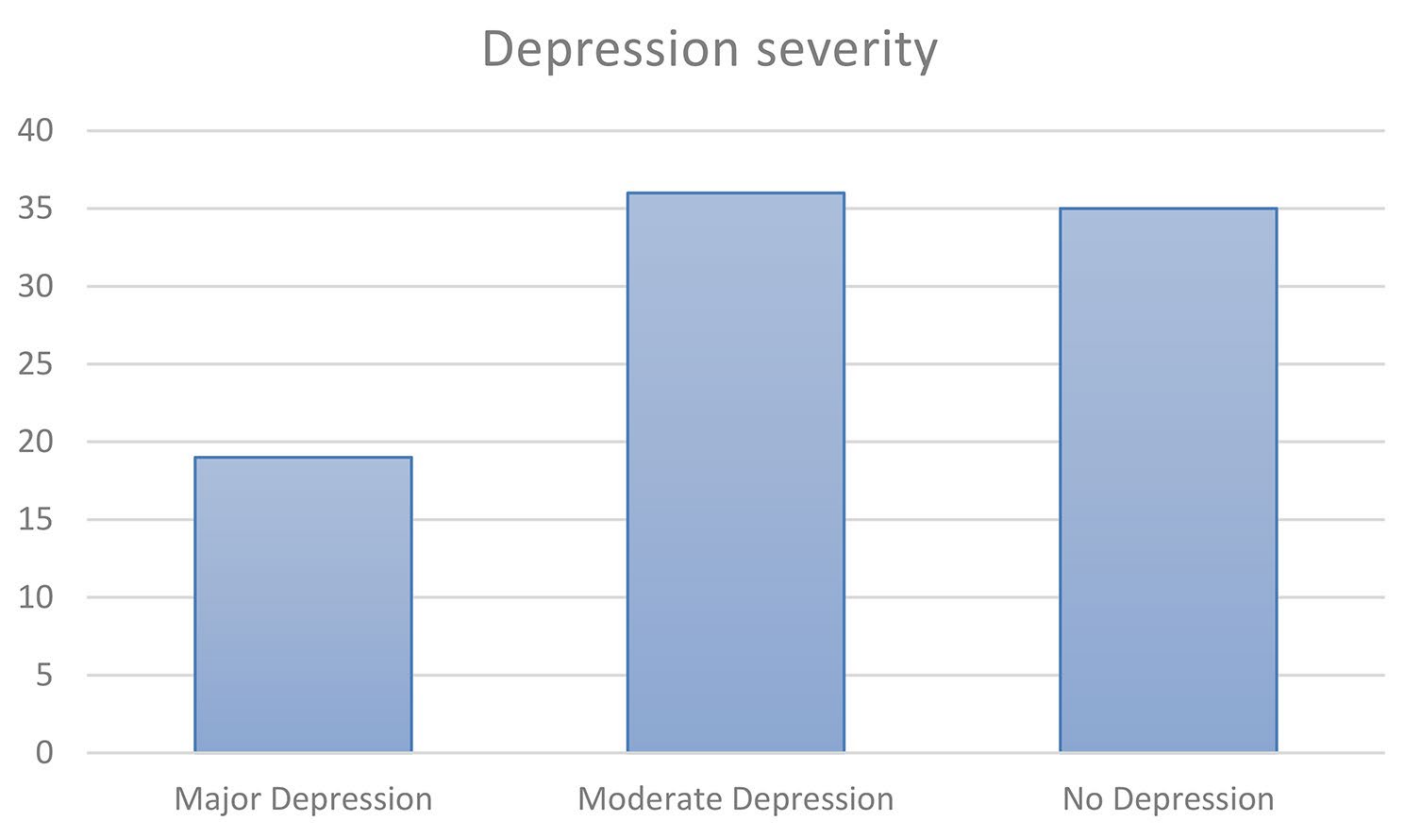
Severity of depression in the study group, according to GDS

The prevalence of depressive disorders in the study group was checked using the GDS method. The mean score on GDS was 6.911 ± 3.9081 (me– 7; IQR 4–9). Out of the study group (*n* = 90), the highest percentage of people had moderate depression symptoms (*n* = 36;40%), followed by those without depression (*n* = 35;38.9%). Scores indicative of major depression symptoms were recorded in 19 respondents (21.1%). The results are presented in Fig. 2.

In stage 1 of the study, we examined whether GDS scores were correlated with the values of the measured blood parameters. As shown in Table 2, a weak negative correlation was observed for fibrinogen (*p* ≤ 0.05).

**Table 2:** Correlation between GDS scores and tested blood parameters

	CRP (mg/L)	Fibrinogen (µg/mL)	IL_10 (pg/mL)	IL_1α (pg/mL)	IL_1β (pg/mL)	IL_6 (pg/mL)	TGF_β1 (pg/mL)	TNF_α (pg/mL)	age (years)
rho	-0.019	-0.215	-0.047	-0.014	0.008	0.049	0.087	-0.046	0.101
*p*-value	0.857	0.042	0.661	0.899	0.938	0.644	0.417	0.666	0.345
FDR	0.938	0.378	0.938	0.938	0.938	0.938	0.938	0.938	0.938
N	90	90	90	90	90	90	90	90	90

Rho- Spearman correlation coefficient; FDR - false discovery rate; N- number of population

When the GDS scores were interpreted according to the adopted classification criteria and analysed in relation to the same blood parameters, no significant relationships were found. Tables 3 and 4.

**Table 3:** Testing for significant variables in the relationship between depression severity and selected blood parameters

Factors	GDS	N	Median	Q1–Q3	min–max	Kruskal-Wallis test; *p*-value	FDR
TNF_α (pg/mL)	MAJOR DEPRESSION	19	8.70	6.96–10.51	5.17–27.04	H = 0.975 *p* = 0.615	0.958
	MODERATE DEPRESSION	36	7.89	5.57–11.66	1.23–69.06		
	NO DEPRESSION	35	8.92	5.89–13.45	2.39–67.37		
TGF_β1 (pg/mL)	MAJOR DEPRESSION	19	19	38,910	27778.50-59460.00	H = 0.793 *p* = 0.673	0.958
	MODERATE DEPRESSION	36	36	35,385	29551.50-44310.00		
	NO DEPRESSION	35	35	39,960	28994.25–47370.00		
CRP (mg/L)	MAJOR DEPRESSION	19	10.29	5.45–16.45	1.50-33.57	H = 3.644; *p* = 0.162	0.958
	MODERATE DEPRESSION	36	4.86	2.29–11.08	1.02–46.29		
	NO DEPRESSION	35	7.91	3.44–13.65	0.74–44.39		
Fibrinogen (µg/mL)	MAJOR DEPRESSION	19	40.89	40.81–41.05	40.73–41.14	H = 2.735 *p* = 0.255	0.958
	MODERATE DEPRESSION	36	40.89	40.73–41.05	4.19–41.14		
	NO DEPRESSION	35	40.97	40.89–41.05	5.83–41.22		
IL_10 (pg/mL)	MAJOR DEPRESSION	19	1.60	1.60–8.22	1.60-129.40	H = 1.0061 *p* = 0.605	0.958
	MODERATE DEPRESSION	36	4.07	1.60-28.45	1.60-631.70		
	NO DEPRESSION	35	3.11	1.60-17.52	1.60–76.20		
IL_1α (pg/mL)	MAJOR DEPRESSION	19	1.10	1.10–1.10	1.10–1.92	H = 0.203 *p* = 0.904	0.958
	MODERATE DEPRESSION	36	1.10	1.10–1.11	1.10-10.19		
	NO DEPRESSION	35	1.10	1.10–1.10	1.10-23.260		
IL_1β (pg/mL)	MAJOR DEPRESSION	19	0.35	0.35–0.35	0.35–293.10	H = 0.445 *p* = 0.799	0.958
	MODERATE DEPRESSION	36	0.35	0.35–0.35	0.35–1355		
	NO DEPRESSION	35	0.35	0.35–0.35	0.35–748.40		
IL_6 (pg/mL)	MAJOR DEPRESSION	19	20.27	12.49–46.09	7.46–437.40	H = 0.086 *p* = 0.958	0.958
	MODERATE DEPRESSION	36	22.36	13.20-51.12	2-668.80		
	NO DEPRESSION	35	27.16	7.82–57.95	2-596		

Q1– First Quartile; Q3– Third Quartile; N- number of population; min-minimum; max-maximum;FDR - false discovery rate

**Table 4:** Testing for significant parameters in the relationship between presence of depression and selected blood parameters

Variable	Depression="Depression”			Depression="No depression”			*p*-value for Mann-Whitney test	FDR
	N	Median	Q1–Q3	N	Median	Q1–Q3		
TNF_α (pg/mL)	55	8.405	6.71–11.30	35	8.924	5.89–13.45	0.617	0.898
TGF_β1 (pg/mL)	55	37,110	29350.50-46327.50	35	39,960	28994.25–47370.00	0.719	0.898
CRP (mg/L)	55	6.38	2.41–11.83	35	7.91	3.45–13.66	0.454	0.898
Fibrinogen (µg/mL)	55	40.89	40.81–41.05	35	40.97	40.89–41.05	0.102	0.898
IL_10 (pg/mL)	55	2.467	1.60-25.39	35	3.107	1.60-17.52	0.806	0.898
IL_1α (pg/mL)	55	1.1	1.10–1.10	35	1.1	1.10–1.10	0.898	0.898
IL_1β (pg/mL)	55	0.35	0.35–0.35	35	0.35	0.35–0.35	0.606	0.898
IL_6 (pg/mL)	55	20.27	13.20–48.00	35	27.16	7.82–57.95	0.769	0.898

Q1– First Quartile; Q3– Third Quartile; N- number of population; FDR - false discovery rate

Then, we analysed whether the levels of the cytokines studied could predict the development of depression. However, the logistic regression model did not show any significant relationships (Table 5).

**Table 5:** Logistic regression for predictors of developing depression

Variable	Coefficient	Std. Error	Wald	P	Odds ratio	95% CI
TNF_α (pg/mL)	-0.00907	0.024748	0.1342	0.7141	0.991	0.9441 to 1.0402
TGF_β1 (pg/mL)	-1.4E-05	1.64E-05	0.6827	0.4086	1	1.0000 to 1.0000
CRP (mg/L)	-0.00295	0.024845	0.01412	0.9054	0.9971	0.9497 to 1.0468
Fibrinogen (µg/mL)	-0.13312	0.14924	0.7957	0.3724	0.8754	0.6533 to 1.1728
IL_10 (pg/mL)	0.019547	0.015589	1.5722	0.2099	1.0197	0.9891 to 1.0514
IL_1α (pg/mL)	-0.32407	0.30289	1.1447	0.2847	0.7232	0.3994 to 1.3094
IL_1β (pg/mL)	0.000507	0.001407	0.1299	0.7185	1.0005	0.9978 to 1.0033

*p*-*p*-value

In the final stage of the study, the parameters measured were examined to determine which could serve as predictors of symptoms of any level of depression (depression *n* = 55). The results are presented in the Table 6. It was established that none of the analyzed parameters are significant predictors of depression within the studied group (*p* = 0.2291).

**Table 6:** Predictors of symptoms of any level of depression

		Depression symptoms (*n* = 55)			
Variable	Coefficient	Std. Error	Odds ratio	95% CI	*P*
TNF-α [pg/ml]	-0.016045	0.024988	0.9841	0.9370 to 1.0335	0.5208
TGF_β1 (pg/mL)	-0.000028501	0.00001703	1	0.9999 to 1.0000	0.0942
Fibrinogen (µg/mL)	0.31362	0.22253	1.3684	0.8847 to 2.1165	0.1587
IL_10 (pg/mL)	0.01234	0.011338	1.0124	0.9902 to 1.0352	0.2764
IL_1β (pg/mL)	0.014684	0.012923	1.0148	0.9894 to 1.0408	0.2558
IL_1α (pg/mL)	0.5412	0.35588	1.7181	0.8553 to 3.4512	0.1283
IL_6(pg/mL)	-0.0024651	0.0016562	0.9975	0.9943 to 1.0008	0.1366
Alzheimer’s disease	20.96395	17320.2784	1,270,000,000	NA	0.999
Parkinson’s disease	0.029617	1.58242	1.0301	0.0463 to 22.9005	0.9851
Coronary disease	0.059827	0.89055	1.0617	0.1853 to 6.0819	0.9464
Hypertension	0.84906	0.66259	2.3375	0.6379 to 8.5655	0.2
Cancer	1.78127	1.37069	5.9374	0.4044 to 87.1666	0.1938
Osteoporosis	0.21687	0.94583	1.2422	0.1946 to 7.9303	0.8186
Stroke	20.00877	8573.99365	489,000,000	NA	0.9981
Myocardial infarction	1.25588	1.59924	3.5109	0.1528 to 80.6717	0.4323

## Discussion

The correlation between elevated levels of inflammatory proteins in the blood and the likelihood of late-life depression remains uncertain. It is worth noting that cytokines may act as intermediaries between immune cells and nerve cells. Within the brain, they have been linked to immunological, neurochemical, neuroendocrine, and behavioural functions [21]. The excessive release of pro-inflammatory cytokines has been postulated to play a role in the development of depression [22]. Pro-inflammatory cytokines interfere with many of the pathophysiological mechanisms that are characteristic of the pathogenesis of depression, such as reduced synaptic plasticity and altered serotonin metabolism [23, 24]. Hayley et al. [25] have advanced the proposition that the activation of neuroinflammatory cascades, mediated by pro-inflammatory cytokines, induces detrimental effects on neurons via the release of oxidative species by microglia. Their hypothesis postulates a potential influence of cytokines on brain-derived neurotrophic factor [26] and serotonin, which may lead to the manifestation of depressive symptoms. Furthermore, these authors have posited that the release of cytokines may indirectly contribute to depression by instigating oxidative processes, which in turn adversely impact neuroplasticity [25].

Over the years, numerous studies have been carried out among older people in different countries around the world in search of a relationship between pro-inflammatory factors and depression. However, the results of these studies are inconsistent.

A study of 358 elderly people in the Netherlands found no statistically significant associations between IL-6 and CRP levels and depression, which was analysed in terms of characteristics including severity, age of onset and type of depression (normal, atypical, melancholic) [27]. Forti et al., in a two-stage study with a 4-year interval on a group of 968 elderly people in Italy, after adjustment for possible confounders and multiple comparisons, also failed to demonstrate the usefulness of the blood inflammatory proteins IL-6, TNF- α, CRP as potential predictors of the development of depression in older age [28]. A meta-analysis by Ng et al., including 34 studies involving a total of 2609 older people with depression and 14,363 controls, found that TNF-α and CRP levels were not significantly different between older people with and without depressive disorders [11]. Our study also found no significant relationships between IL-6, CRP and TNF-α and depression.

On the other hand, in a cross-sectional study conducted in Brazil, an assessment of the relationship between IL-1β and the incidence of depression in older age found higher levels of this pro-inflammatory factor in people with depression compared with controls. After dividing the patients with depression into late-onset and early-onset groups, it was found that patients in the second group had the highest levels of IL-1β. The authors suggest that an increased pro-inflammatory state may play a role in the physiopathology of depression in older people [29]. Thomas et al. [30] and Torres et al. [31] showed elevated IL-1β levels in late-onset depression patients compared with controls. Charlton et al., in a study involving a group of US seniors, showed that pro-inflammatory cytokines (IL-1β, TNF-α and IL-6) were higher in late-life depression compared to healthy older adults [32]. In addition, a study conducted in Greece suggested an association between elevated levels of IL-6 and depressive symptoms in older people [33]. A study from Poland found significantly higher levels of both IL-6 and CRP in study participants with depression than in those without [34]. In turn, Martinez-Cengotitabengoa et al. conducted a systematic review based on a qualitative analysis of six studies and found that elevated levels of peripheral IL-6 and TNF-α may indicate vulnerability to depression in late life [35]. Penninx et al. showed that not only are the levels of inflammatory cytokines elevated in patients with major depression, but there is also a link between elevated levels of CRP, IL-6 and TNF-α and depressed mood in a community-based sample of older people [36].

Research findings also indicate that both major depression and depressive symptoms are associated with elevated serum concentrations of inflammatory mediators, including acute-phase proteins such as CRP and fibrinogen [37–39], and these findings are not restricted to older age groups. In a study combining data from two large population-based studies in Copenhagen, involving 73,367 men and women aged 20–100 years, elevated fibrinogen levels were associated with psychological distress, antidepressant use and hospitalisation for depression [40]. In the present study, we showed a weak negative correlation between GDS scores and fibrinogen levels, but when GDS scores were interpreted according to the adopted classification criteria this relationship was not confirmed.

TGF-β1 is a factor with anti-inflammatory and neuroprotective properties against amyloid-β (Aβ)-induced neuronal degeneration and is crucial for memory formation and synaptic plasticity. Reduced plasma TGF-β1 levels have been observed in patients with major depression, correlating with the severity of depressive symptoms and contributing significantly to treatment resistance in major depression [41]. This is not confirmed by the present study. In animal models of depression, increased levels of pro-inflammatory cytokines, such as IL-1β and TNF-α, and decreased levels of anti-inflammatory cytokines, such as IL-10 and TGF-β1, have been observed in the hippocampus and cortex [42].

The results of the previously mentioned meta-analysis by Ng et al. showed significantly higher levels of IL-1β and IL-6 in older people with diagnosed depression compared to those without depressive disorders [11]. In our study, there were no statistically significant correlations between the levels of the cytokines studied and the severity or development of depression.

As we age, our effectiveness tends to decline. If our lives have been successful, we derive satisfaction from what we have achieved and how it has impacted us. However, if we feel that we have not achieved our life goals and are dissatisfied with life, we may experience a sense of despair leading to depressive disorders [43]. In a study by the authors, 35 people showed no symptoms of depression. In the article by Aiello A. et al., the case of a 100-year-old resident of Sicily who also showed no symptoms of depression was described. Research focusing on centenarians portrays them as the optimal model of aging. In situations where age-related diseases occur between the ages of 80 and 99, they are termed “delayed”; if diseases occur before the age of 80, they are termed “survivors”; and if they reach the age of 100 without any diseases, they are referred to as “escapers” [44].Uncertainties and conflicting research findings regarding the links between depression and levels of pro-inflammatory factors highlight the need for further research, potentially as part of a comprehensive exploration of the interplay between oxidative damage and inflammation in the pathophysiology of geriatric depression.

## Conclusions

Inflammatory parameters do not appear to predict the development of depressive disorders in people over 90 years of age.

### Limitations

One limitation of the study was the small group size. It arises from the fact that the study included individuals who were in logical contact and expressed informed consent to participate in the study. Further research should be considered to increase the sample size. The Geriatric Depression Scale is one of the more commonly used tools to screen for depression in the elderly. It is a self-assessment scale which means that it relies on the respondent’s honesty, which is subjective.

## Data Availability

The datasets used and/or analysed during the current study are available from the first author on reasonable request.

## References

[1] Sharifi S, Babaei Khorzoughi K, Rahmati M (2024). The association between intergenerational relationships and depression among older adults: a comprehensive systematic literature review. Arch Gerontol Geriatr.

[2] UN: Population Division. World population projected to reach 9.7 billion by 2050. *Department of Economic and Social Affairs*. http://www.un.org/en/development/desa/news/population/2015-report.html (2015).

[3] World Health Organisation & Alzheimer’s Disease International. Dementia: A Public Health Priority. *World Health Organization*. http://www.who.int/mental_health/publications/dementia_report_2012/en/ (2012).

[4] Ho RC, Ho EC, Tai BC, Ng WY, Chia BH (2014). Elderly suicide with and without a history of suicidal behavior: implications for suicide prevention and management. Archives Suicide Res.

[5] Puri B, Hall A, Ho R. Revision notes in Psychiatry, Third Edition. Taylor & Francis; 2013.

[6] Loh AZ, Tan JS, Zhang MW, Ho RC (2016). The global prevalence of anxiety and depressive symptoms among caregivers of stroke survivors. J Am Med Dir Assoc.

[7] Zhang MW, Ho RC, Cheung MW, Fu E, Mak A (2011). Prevalence of depressive symptoms in patients with chronic obstructive pulmonary disease: a systematic review, meta-analysis and meta-regression. Gen Hosp Psychiatry.

[8] Liu Y, Ho RC, Mak A, Interleukin (2012). IL)-6, tumour necrosis factor alpha (TNF-α) and soluble interleukin-2 receptors (sIL-2R) are elevated in patients with major depressive disorder: a meta-analysis and meta-regression. J Affect Disord.

[9] Maes M, Springer. 1999. Major depression and activation of the inflammatory response system, in Cytokines, stress, and depression; pp. 25–46. 10.1007/978-0-585-37970-8_2 10442165

[10] Lu Y (2012). Prevalence of anxiety and depressive symptoms in adolescents with asthma: a meta-analysis and meta‐regression. Pediatr Allergy Immunol.

[11] Ng A, Tam WW, Zhang MW, Ho CS, Husain SF, McIntyre RS, Ho RC, IL-1β. IL-6, TNF- α and CRP in Elderly patients with Depression or Alzheimer’s disease: systematic review and Meta-analysis. Sci Rep. 2018;8(1):12050. 10.1038/s41598-018-30487-6. PMID: 30104698; PMCID: PMC6089986. 10.1038/s41598-018-30487-6 PMC6089986 30104698

[12] Orsolini L, Pompili S, Tempia Valenta S, Salvi V, Volpe U (2022). C-Reactive protein as a biomarker for major depressive disorder?. Int J Mol Sci.

[13] Wu X, Dai B, Yan F, Chen Y, Xu Y, Xia Q, Zhang X, Serum Cortisol (2022). Nesfatin-1, and IL-1β: potential diagnostic biomarkers in Elderly patients with treatment-resistant depression. Clin Interv Aging.

[14] Haapakoski R, Mathieu J, Ebmeier KP, Alenius H, Kivimäki M (2015). Cumulative meta-analysis of interleukins 6 and 1β, tumour necrosis factor α and C-reactive protein in patients with major depressive disorder. Brain Behav Immun.

[15] Capuron L, Miller AH (2011). Immune system to brain signaling: neuropsychopharmacological implications. Pharmacol Ther.

[16] Musil R, Schwarz MJ, Riedel M, Dehning S, Cerovecki A, Spellmann I, Arolt V, Muller N (2011). Elevated macrophage migration inhibitory factor and decreased transforming growth factor-beta levels in major depression–no influence of celecoxib treatment. J Affect Disord.

[17] Rush G, O’Donovan A, Nagle L, Conway C, McCrohan A, O’Farrelly C, Lucey JV, Malone KM (2016). Alteration of immune markers in a group of melancholic depressed patients and their response to electroconvulsive therapy. J Affect Disord.

[18] Maes M (1999). Major depression and activation of the inflammatory response system Adv. Exp Med Biol.

[19] Myint AM, Leonard BE, Steinbusch HW, Kim YK (2005). Th1, Th2, and Th3 cytokine alterations in major depression. J Affect Disord.

[20] Xie Z, Li C, Xing Z, Zhou W, Xie S, Li M, Zhou Y (2021). Relationship between serum fibrinogen level and depressive symptoms in an Adult Population with spinal cord Injury: a cross-sectional study. Neuropsychiatr Dis Treat.

[21] Kronfol Z, Remick D (2000). Cytokines and the brain. Implications for clinical psychiatry. Am J Psychiatry.

[22] O’Brien SM, Scott LV, Dinan TG (2004). Cytokines: abnormalities in major depression and implications for pharmacological treatment. Human Psychopharmacol.

[23] Remus JL, Dantzer R. Inflammation models of depression in rodents: relevance to psychotropic drug discovery. Int J Neuropsychopharmacol. 2016;19(9). 10.1093/ijnp/pyw028. 10.1093/ijnp/pyw028 PMC5043641 27026361

[24] Maes M, Nowak G, Caso JR, Leza JC, Song C, Kubera M, Klein H, Galecki P, Noto C, Glaab E, Balling R, Berk M (2016). Toward Omics-Based systems biomedicine, and path and drug discovery methodologies for depression-inflammation research. Mol Neurobiol.

[25] Hayley S, Poulter MO, Merali Z, Anisman H (2005). The pathogenesis of clinical depression: stressor- and cytokine-induced alterations of neuroplasticity. Neuroscience.

[26] Murphy PG, Borthwick LA, Altares M, Gauldie J, Kaplan D, Richardson PM (2000). Reciprocal actions of interleukin-6 and brain derived neurotrophic factor on rat and mouse primary sensory neurons. Eur J Neurosci.

[27] Vogelzangs N, Comijs HC, Oude Voshaar RC, Stek ML, Penninx BW. Late-life depression symptom profiles are differentially associated with immunometabolic functioning. Brain Behav Immun. 2014;41:109– 15. 10.1016/j.bbi.2014.05.004. Epub 2014 May 13. PMID: 24838021. 10.1016/j.bbi.2014.05.004 24838021

[28] Forti P, Rietti E, Pisacane N, Olivelli V, Mariani E, Chiappelli M, Licastro F, Ravaglia G. Blood inflammatory proteins and risk of incident depression in the elderly. Dement Geriatr Cogn Disord. 2010;29(1):11–20. 10.1159/000261644. PMID: 20068306. 10.1159/000261644 20068306

[29] Diniz BS, Teixeira AL, Talib L, Gattaz WF, Forlenza OV. Interleukin-1beta serum levels is increased in antidepressant-free elderly depressed patients. Am J Geriatr Psychiatry. 2010;18(2):172-6. 10.1097/JGP.0b013e3181c2947f. PMID: 20104073. 10.1097/JGP.0b013e3181c2947f 20104073

[30] Thomas AJ, Davis S, Morris C (2005). Increase in interleukin-1 beta in late-life depression. Am J Psychiatry.

[31] Torres KC, Lima GS, Fiamoncini CM, Rezende VB, Pereira PA, Bicalho MA, Moraes EN, Romano-Silva MA (2014). Increased frequency of cluster of differentiation 14 (CD14+) monocytes expressing interleukin 1 beta (IL-1β) in Alzheimer’s disease patients and intermediate levels in late-onset depression patients. Int J Geriatr Psychiatry.

[32] Charlton RA, Lamar M, Zhang A, Ren X, Ajilore O, Pandey GN, Kumar A (2018). Associations between pro-inflammatory cytokines, learning, and memory in late-life depression and healthy aging. Int J Geriatr Psychiatry.

[33] Dimopoulos N, Piperi C, Psarra V, Lea RW, Kalofoutis A (2008). Increased plasma levels of 8-iso-PGF2alpha and IL-6 in an elderly population with depression. Psychiatry Res.

[34] Nadrowski P (2016). Associations between cardiovascular disease risk factors and IL-6 and hsCRP levels in the elderly. Exp Gerontol.

[35] Martínez-Cengotitabengoa M, Carrascón L, O’Brien JT, Díaz-Gutiérrez MJ, Bermúdez-Ampudia C, Sanada K, Arrasate M, González-Pinto A (2016). Peripheral inflammatory parameters in late-life depression: a systematic review. Int J Mol Sci.

[36] Penninx BW, Kritchevsky SB, Yaffe K, Newman AB, Simonsick EM, Rubin S (2003). Inflammatory markers and depressed mood in older persons: results from the Health, Aging and Body Composition Study. Biol Psychiatry.

[37] Ladwig KH, Marten-Mittag B, Lowel H, Doring A, Koenig W (2003). Influence of depressive mood on the association of CRP and obesity in 3,205 middle aged healthy men. Brain Behav Immun.

[38] Joynt KE, Whellan DJ, O’Connor CM (2004). Why is depression bad for the failing heart? A review of the mechanistic relationship between depression and heart failure. J Card Fail.

[39] Corcos M, Guilbaud O, Hjalmarsson L, Chambry J, Jeammet P (2002). Cytokines and depression: an analogic approach. Biomedecine Pharmacotherapy.

[40] Wium-Andersen MK, Ørsted DD, Nordestgaard BG (2013). Elevated plasma fibrinogen, psychological distress, antidepressant use, and hospitalization with depression: two large population-based studies. Psychoneuroendocrinology.

[41] Caraci F, Spampinato SF, Morgese MG, Tascedda F, Salluzzo MG, Giambirtone MC, Caruso G, Munafò A, Torrisi SA, Leggio GM, Trabace L, Nicoletti F, Drago F, Sortino MA, Copani A (2018). Neurobiological links between depression and AD: the role of TGF-β1 signaling as a new pharmacological target. Pharmacol Res.

[42] You Z, Luo C, Zhang W, Chen Y, He J, Zhao Q, Zuo R, Wu Y (2011). Pro- and anti-inflammatory cytokines expression in rat’s brain and spleen exposed to chronic mild stress: involvement in depression. Behav Brain Res.

[43] Kida H, Niimura H, Eguchi Y, Suzuki K, Shikimoto R, Bun S, Takayama M, Mimura M (2023). Relationship between life satisfaction and psychological characteristics among Community-Dwelling Oldest-old: focusing on Erikson’s Developmental stages and the big five personality traits. Am J Geriatric Psychiatry.

[44] Aiello A, Accardi G, Aprile S (2021). Pro-inflammatory status is not a limit for longevity: case report of a sicilian centenarian. Aging Clin Exp Res.

